# Multicentre randomized controlled trial of structured transition on diabetes care management compared to standard diabetes care in adolescents and young adults with type 1 diabetes (Transition Trial)

**DOI:** 10.1186/1471-2431-13-163

**Published:** 2013-10-09

**Authors:** Tamara Spaic, Jeff L Mahon, Irene Hramiak, Nicole Byers, Keira Evans, Tracy Robinson, Margaret L Lawson, Janine Malcolm, Ellen B Goldbloom, Cheril L Clarson

**Affiliations:** 1 St. Joseph’s Health Care, London, ON, Canada; 2 Department of Medicine, Western University, London, ON, Canada; 3 Department of Epidemiology and Biostatistics, Western University, London, ON, Canada; 4 Children’s Hospital, London Health Sciences Centre, London, ON, Canada; 5 Department of Paediatrics, Western University, London, ON, Canada; 6 Department of Sociology, Western University, London, ON, Canada; 7 Children’s Hospital of Eastern Ontario, Ottawa, ON, Canada; 8 The Ottawa Hospital, Ottawa, ON, Canada; 9 Division of Endocrinology and Metabolism, University of Ottawa, Ottawa, ON, Canada

**Keywords:** Transition care, Adolescents and young adults, Transition intervention, Chronic illness, Type 1 diabetes, Healthcare systems

## Abstract

**Background:**

Transition from pediatric to adult diabetes care is a high risk period during which there is an increased rate of disengagement from care. Suboptimal transition has been associated with higher risks for acute and chronic diabetes-related complications. The period of emerging adulthood challenges current systems of healthcare delivery as many young adults with type 1 diabetes (T1D) default from diabetes care and are at risk for diabetes complications which are undetected and therefore untreated. Despite the importance of minimizing loss to follow-up there are no randomized control trials evaluating models of transition from pediatric to adult diabetes care.

**Methods/Design:**

This is a multicentre randomized controlled trial. A minimum of 188 subjects with T1D aged between 17 and 20 years will be evaluated. Eligible subjects will be recruited from three pediatric care centres and randomly assigned in a 1:1 ratio to a structured transition program that will span 18 months or to receive standard diabetes care. The structured transition program is a multidisciplinary, complex intervention aiming to provide additional support in the transition period. A Transition Coordinator will provide transition support and will provide the link between pediatric and adult diabetes care. The Transition Coordinator is central to the intervention to facilitate ongoing contact with the medical system as well as education and clinical support where appropriate. Subjects will be seen in the pediatric care setting for 6 months and will then be transferred to the adult care setting where they will be seen for one year. There will then be a one-year follow-up period for outcome assessment. The primary outcome is the proportion of subjects who fail to attend at least one outpatient adult diabetes specialist visit during the second year after transition to adult diabetes care. Secondary outcome measures include A1C frequency measurement and levels, diabetes related emergency room visits and hospital admissions, frequency of complication screening, and subject perception and satisfaction with care.

**Discussion:**

This trial will determine if the support of a Transition Coordinator improves health outcomes for this at-risk population of young adults.

**Trial registration:**

Trial Registration Number: NCT01351857

## Background

Transition from pediatric to adult medical care of adolescents and young adults with T1D has been a challenging issue for decades. A recent American Diabetes Association (ADA) Position Statement [1] highlighted that transition from pediatric to adult diabetes care is a high risk period during which there is an increased rate of disengagement from care. Suboptimal transition has been associated with higher risks for acute and chronic diabetes-related complications. Yet, the question of how best to transition young T1D patients remains unanswered, in part due to lack of randomized control trials evaluating models of transition from pediatric to adult diabetes care.

### Factors associated with suboptimal glycemic control

Many challenges are faced during adolescence by young adults who are establishing personal identity, sexual behaviors and increasing independence. It is a period of transition regardless of their health status due to the increasing influence of peers combined with other contributing societal factors. For anyone coping with the daily demands of managing a chronic disease, young adulthood is even more complex. Diabetes control may deteriorate significantly during this period due to multiple factors including: physiological insulin resistance associated with hormonal changes of puberty, psychosocial distress, risk taking behavior, intentional insulin omission for weight loss or attention, and eating disorders [2,3]. Adolescence is therefore a particularly vulnerable period in diabetes care. In addition, one of the major changes that occur in a young person’s life during this time is the transition from pediatric to adult medical care. Emerging adults may have limited experience with basic tasks often routinely managed by parents, such as scheduling their own medical appointments and maintaining prescribed medical supplies [4]. The transition from pediatric to adult care also may coincide with a loss of health insurance coverage and an increase in financial barriers to healthcare access [5].

### Impact on glycemic control and diabetes related complications

Transition of care has a major impact on blood glucose control and disease outcomes in patients with T1D [6-8]. In the first year of transition 11- 41% of T1D patients drop out of adult medical care [9-11] and 46% report difficulties with the transition process [9]. In a retrospective study, 27% of patients were not followed in an Adult Diabetes Service three years after the last pediatric visit [12].

Individuals who are lost to follow-up have higher A1C values during the 2 years prior to transition of care [13]. This suggests that this poorly controlled population is especially vulnerable to disengagement. Risk factors for poor compliance after transfer to adult diabetes care include female gender, no college degree, poor glycemic control and fewer diabetes care visits in the year prior to transfer [14,15]. The following barriers to successful transition have been identified by individuals with T1D: abrupt transfer of care, lack of accessibility of adult-care services, lack of coordination between different disciplines involved in the care and lengthy waiting periods [14,16].

The impact of loss to follow-up on the health of transitioning youth is significant. Among those lost to medical follow-up, the mean A1C is on average 1.5% higher than those who maintain medical follow-up [13,17-20]. Background retinopathy increases from 5% to 29% [17] and nephropathy by 17% [21]. A 38% pregnancy loss was reported in a group that had no intervention during transition, compared to none in a group who used a central, coordinated navigation service for care, education, and support [22]. Furthermore, diabetes related hospitalization rates increase significantly from 7.6 to 9.5 cases per 100 patient-years in the two years after transition to adult care [21]. UK data for the 20–29 age group show that mortality is increased three fold in men and six fold in women when compared with the general population [15]. The major causes for mortality are acute complications, with 68% of diabetes–related deaths being due to diabetic ketoacidosis (DKA) or hypoglycemia [15].

### Transition interventions

Transition support programs improve the quality of diabetes care in young adults with T1D. A study on the impact of a transition education program at a Toronto diabetes centre reported that the implementation of this program was associated with a decrease in the proportion of patients lost to follow-up from 24% to 7% [10,23]. Studies assessing the role of a transition coordinator have found 0.13% lower A1C levels per visit for the first 4 visits when a transition coordinator was consistently involved with the care of young adult T1D patients [24]. Other studies have demonstrated that a structured transition which includes a collaborative effort between adult and pediatric endocrinology results in a 23% decrease in rates of loss to follow-up for up to three years after transition [12]. There was also a significant reduction in admission rates with DKA to approximately 2/3 of the admission rates prior to the program [24]. The number of eye and feet examinations, and microalbuminuria testing were significantly higher in the structured transition group [12,21]. A recent systematic review on effectiveness of various transitional programs identified patient education programs and joint pediatric/adult clinics or specific young adult clinics as services that may improve outcomes in emerging adults with T1D. However, as the authors suggested, the comparative benefit of different components of these complex interventions is not yet clear. It is noted that successful programs also include a transition coordinator role [25]. Finally, structured transition programs have been found to be feasible and acceptable by young T1D adults [9].

### Transition trial

#### ***Aim of the study***

The overall goal of the study is to determine if a structured transition program for adolescents and young adults with T1D improves diabetes clinic attendance and management as well as glycemic control after transition from pediatric to adult diabetes care.

#### ***Study objectives***

The primary objective of the study is to test the hypothesis that the proportion of young adult T1D patients who fail to attend regular diabetes care during the first year after completion of a structured diabetes transition program will decrease when compared to the proportion of non-attendance of those patients receiving standard care. The secondary objectives of the study are to compare the frequency of routine diabetes testing (A1C, microalbuminuria, lipid profile, and retinal exam) as well as rates of hospitalizations for diabetes related problems (DKA and hypoglycemia), and patient satisfaction with the transition process between the groups.

## Methods/Design

### Design

A multicentre, randomized, single-blind controlled trial is being conducted in two tertiary centres (St Joseph’s Health Care and Children’s Hospital, London Health Sciences Centre in London; Children’s Hospital of Eastern Ontario and The Ottawa Hospital in Ottawa) and a secondary centre (Trillium Health Partners, in Mississauga, Ontario). A minimum of 188 subjects are being randomly assigned in a 1:1 ratio to a structured transition program that spans 18 months or to receive standard diabetes care. The structured transition program is a multidisciplinary, complex intervention designed to provide additional support in the transition period. Central to the program is a Transition Coordinator who provides transition support and is the link between pediatric and adult diabetes care. In addition, the Transition Coordinator offers transitional education and clinical support where appropriate. Subjects are seen in the pediatric care setting for 6 months and then transferred to the adult care setting where they are seen for one year. There will then be a one-year follow-up period for outcome assessment. This study has been approved by each clinical site’s local institutional review board (London REB 17892, Ottawa REB 12/11E and 20120169-01H and Mississauga REB 518). Informed consent is obtained from all study participants based on a template provided by the study group (each approved by the local ethics review board) and centrally monitored by the JDRF Canadian Clinical Trial Network (JDRF CCTN).

### Participants

Subjects with T1D who are between ages 17 and 20 years are recruited from the tertiary and secondary specialized pediatric diabetes clinics in the three participating centres in London, Mississauga, and Ottawa. Only residents of Ontario are eligible since the outcomes are to be determined using the large administrative database available only for residents of this province. All diabetes patients scheduled for a visit with the pediatric diabetes team who are approaching transition age (range 17 to 20) are eligible. However, readiness for transition is not assessed formally as part of this study and is at the discretion of the investigators to determine the most appropriate age for transition according to standard current clinical practice. Factors considered are: future career plans, social situation, and geographic relocation. For example an approximate time for transition coincides with completion of high school which in Ontario ranges from age 17 to 19 years. Subjects are included only if able to independently manage their diabetes and those with an intellectual disability requiring caregiver assistance with diabetes management are not eligible for the study. Subjects with ongoing medical issues that interfere with diabetes care and glycemic control, such as high dose steroid treatment or active cancer treatment, are not eligible either. To allow for adjustment to the diagnosis and minimize the impact of residual insulin secretion on glycemic control, subjects are included only if diagnosed with T1D for at least a year.

### Inclusion criteria

1. Established T1D diagnosis for a minimum of one year.

2. Between the ages of 17 and 20 years.

3. At least 1 visit during the previous year with the pediatric endocrinologist at one of the three participating Diabetes Clinics (aim is to minimize non-adherence with the intervention).

4. Ability to participate in all aspects of this clinical trial.

5. Written informed consent/assent must be obtained and documented.

6. Resident of Ontario.

### Exclusion criteria

1. Pregnant or lactating females or intent to become pregnant during the next 3 years.

2. Condition(s) which in the opinion of the investigator may interfere with the subject’s ability to participate in the study.

3. Prior enrolment in the current study.

4. Prior enrolment of a sibling in the current study.

5. Current participation in another clinical trial or participation in another clinical trial in the 6 months prior to enrolment.

### Sample size and statistical analysis

The outcome measure used to calculate sample size is the proportion of subjects who fail to attend diabetes clinic visits during the second year after transfer to adult care. A 60% relative reduction in non-attendance rate is considered clinically important. The drop out rate, defined as non attendance at adult diabetes clinic during the previous year, for young adults transferred from pediatric to adult diabetes care within the London sites between January 2005 and December 2008 was determined to be 28%, consistent with the literature (unpublished observation). Assuming the non-attendance rate in the control group to be 28% (to detect an absolute difference of 16% (i.e., 28% non-attendance rate in the control group compared to 12% in the intervention group), a total of 188 subjects (94 per group) are required to provide 80% power at the 0.05 level of significance. The sample size calculation did not account for loss to follow-up as this is the primary outcome. The primary analysis will be based on comparison of subjects in the two treatment groups who attend 0, 1, and 2 sessions during the one year follow- up period after completion of the intervention. The Cochran-Mantel-Haenszel mean score test will be used. A 2-sided probability of type 1 error of 0.05 will be declared statistically significant. To control for covariates of interest, the proportional odds model will be adopted. Multilevel growth curve modeling will be used to analyze the glycemic control measurements. Multiple linear regression and logistic regression will be applied to control for covariates of interest where applicable. Reporting of the trial will follow the CONSORT guidelines [26,27].

### Study procedures

#### ***Recruitment***

Eligible patients are identified by the local pediatric diabetes clinic staff. The local investigators introduce the study where appropriate and provide a letter of information to all eligible patients If the prospective participant agrees to be approached, the Research Assistant makes contact during the clinic or within a few weeks to answer any questions or concerns regarding the study. If the subject agrees to participate, informed consent is obtained at the time or at the next routine pediatric clinic visit. Specific targets were set to recruit 40% of participants in each of the tertiary centres and 30% in the secondary setting. No targets were specified regarding gender or ethnicity. The recruitment goal is for 200 patients to be enrolled by January 2014.

#### ***Baseline assessment***

Once consent has been obtained, the baseline assessment is completed as part of the initial visit. Baseline characteristics collected are: age, gender, ethnicity, level of education, family structure, distance from the treatment centre, smoking and alcohol use, comorbid conditions, concomitant medications, and family history of diabetes. In addition, baseline assessment includes detailed initial medical history, measurement of weight and height, blood pressure, capillary A1C for randomization procedure, centralized venous A1C, insulin use, clinic attendance, and completion of baseline patient satisfaction questionnaires. For a complete list of study measures please see the "Summary of Study Measures" section.

#### ***Summary of study measures***

Measures:

1. Historical

a. Sociodemographic: age, ethnicity, sex, level of education, persons living with participants/family structure, distance from the treating centre, and consent to access the Institute for Clinical Evaluative Studies (ICES) data during the study period to determine participant use of the healthcare system

a. Medical history: detailed initial medical history; family history of diabetes-related complications, social habits (smoking, alcohol, illicit drug use), follow-up interim history with focus on hospital visits for hypoglycemia and DKA

a. Insulin dosage and method of delivery

a. Frequency of medical care (retinal, monofilament, lipid profile testing and microalbumin to creatinine ratio)

a. Concomitant medications: all longstanding therapies, with the emphasis placed on insulin therapy

a. Questionnaires (Diabetes Quality of Life Measure; Client Satisfaction Questionnaire; Diabetes Distress Scale)

2. Physical examination measures

a. Anthropometric measurements: height, weight, and BMI

a. Blood Pressure

a. Systems physical examination: general survey, skin, head, neck, chest, heart, abdomen, musculoskeletal/extremities, and neurologic (including lower extremity monofilament testing)

3. Laboratory measures

a. A1C (centralized)

a. Fasting lipid profile

a. Urine pregnancy test (females)

#### ***Randomization***

**Figure 1:**
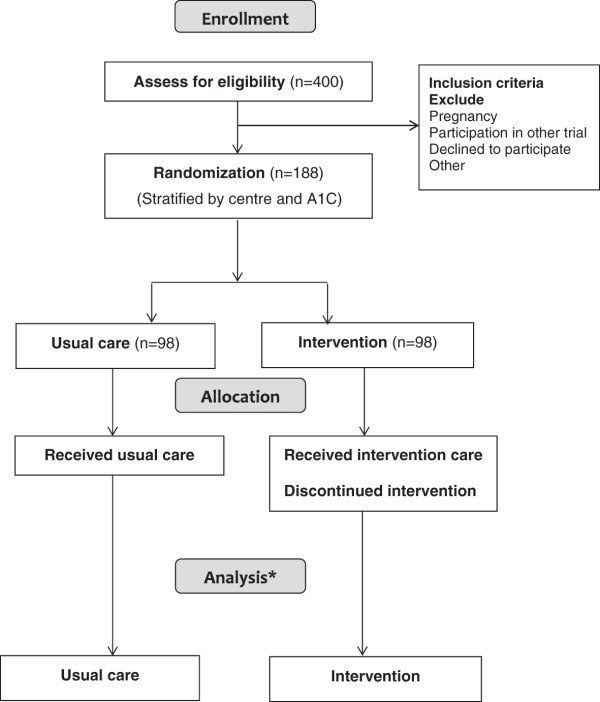
**Transition flow diagram.** *Losses to follow up are considered the primary outcome.

Eligible subjects who have signed informed consent are randomly assigned in a 1:1 ratio to either a structured transition program or to receive standard diabetes care. The randomization schedule is computer generated in variable blocks stratified by 1) centre and 2) the visit 1 A1C (< 8.5% or ≥ 8.5%). The Research Assistant informs the participant of the Randomization group assigned. If the participant is randomized to the Intervention Group, the Research Assistant notifies the Transition Coordinator. Figure 1. provides a flow chart of participants through the study.

#### ***Blinding***

Due to the nature of the intervention it is not possible to blind participants and members of the interdisciplinary team to group allocation. However, data analysis personnel and outcome assessors are blinded to the group assignment. To minimize the possible bias, the allocation sequence is concealed until the subject qualifies for the study and the intervention is assigned. A potential source of bias is treatment cross contamination which is minimized by not permitting any contact between the Transition Coordinator and the control group for the duration of the study, having the consent process conducted by the Research Assistant for both groups, and aiming for physicians to provide the same standard of care to both groups by not being involved in the delivery of the intervention or discussions of the implications. The Canadian Diabetes Association 2008 clinical practice guidelines for the management of T1D patients will be followed in both groups [28].

#### ***Transition intervention***

Subjects randomized to the intervention group are enrolled in the transition program.

The structured transition program is a multidisciplinary, complex intervention aiming to provide additional support during the transition period. The intervention lasts 18 months, 6 months in pediatric care and 12 months in adult care. Table 1 illustrates the study timeline.

**Table 1:** Transition study timeline

**Type of care**	**Pediatric**			**Adult**			**Follow-up**	
	**Visit 1**	**Visit 2**	**Visit 3**	**Visit 4**	**Visit 5**	**Visit 6**	**Visit 7**	**Visit 8**
**Month**	0	3	6	10	14	18	23	28
**Control**	Pediatric Team	Pediatric Team*	Pediatric Team	Adult Endo, DEC	Adult Endo	Adult Endo	Adult Endo	Adult Endo
**Intervention**	Pediatric Team	Pediatric Team*	Pediatric Team	Adult Endo, DEC	Adult Endo	Adult Endo	Adult Endo	Adult Endo
	+ TC	+ TC	+ TC	+ TC	+ TC	+TC		

TC – Transition Coordinator, DEC – Diabetes Education Centre, Endo – Endocrinology clinic.

^*^Referral to Adult Endocrinology Clinic and Diabetes Education Centre takes place.

The Transition program is introduced at least six months prior to scheduled transfer to adult care. At visit 2, three months prior to the last pediatric visit, a referral is made to a local adult diabetes specialist. At those centres where standard practice includes referral to the local diabetes education centre, this is also done at this visit. After six months of the intervention in the pediatric setting, subjects are transitioned to adult diabetes care as per the current practice standard. Subjects are seen in adult care four months from the last pediatric visit. The intervention continues for one year in the adult setting. The Transition Coordinator is a Certified Diabetes Educator (CDE) or CDE prepared and is central to the intervention providing education and clinical support and continuity between pediatric and adult diabetes care.

The role of the Transition Coordinator is to:

• Attend pediatric visits 1, 2, and 3 and adult clinic visits 4, 5, and 6.

• Maintain contact with participants by phone, text, or e-mail.

• Facilitate support for insulin adjustments and sick day/hypoglycemia management during regular hours.

• Send reminders for clinic appointments.

• Reschedule missed appointments, ideally within four weeks.

• Assess needs and facilitate referrals to other services, e.g., psychology, social work, dietitian.

• Provide educational material (handouts, booklets etc.).

• Encourage participants to maintain contact with the family physician.

The Transition Coordinator also provides information and material on the differences in the structure of adult and pediatric diabetes care (e.g., absence of point of care testing for A1C, separate appointments required to follow with members of the interdisciplinary healthcare team in one of the centres, etc.). Age related themes and concerns (body image, sexuality, birth control, drinking, etc.) are addressed and written information provided. Subjects randomized to the control group receive the current standard of pediatric diabetes care. The diabetes interdisciplinary healthcare team differs from the intervention group only by the exclusion of the Transition Coordinator. The team structure is otherwise unchanged.

The Transition Coordinator has no contact with the control group throughout the duration of the study. Within three months following randomization, subjects in the control group are referred to the adult diabetes specialist in the same way as subjects in the intervention group. Subjects in the control group have full access to any education programs and services on transition provided in the community. They are given the opportunity to attend any established transition information session which is part of the standard diabetes care of adolescents and young adults at each centre.

### Measures

#### ***Primary outcome***

The primary outcome is the proportion of subjects who fail to attend at least one outpatient adult diabetes specialist visit during the second year after transition to adult diabetes care.

#### ***Secondary outcomes***

The secondary objectives of the study are:

1. To compare the frequency of A1C testing in the intervention group (transition program) and the control (standard care) group.

2. To compare the mean A1C levels in the intervention and the control groups.

3. To compare the frequency of testing for microalbuminuria, lipid profile, foot, and retinal examinations between the two groups.

4. To compare the rates of diabetes related emergency room visits and hospitalizations for DKA and hypoglycemia in the two groups.

5. To compare the patient satisfaction and perception of the care during the transition period using self-administered questionnaires.

#### ***Adverse events and safety***

Any occurrence with a serious outcome must be reported to CRO Robarts Clinical Trials within 24 hours of learning about the event. Due to the nature of the intervention, it is not expected that serious adverse events related to the intervention will occur. However, adverse events will be collected from the time of signing the Informed Consent. The following adverse events will be recorded in the subject’s medical records and on the case report form:

• Any medical occurrences requiring medical intervention.

• Any action or outcome (e.g., hospitalization, discontinuation of therapy, etc.) will also be recorded for each adverse event.

## Discussion

The role of the Transition Coordinator is the fundamental intervention in this trial, providing a link between pediatric and adult care and ongoing support during the first year after transfer from pediatric care. To date, there are no studies that have directly compared various transition interventions. The intervention in this trial was selected based on evidence from observational studies showing improvement in clinical outcomes [24], and as it is anticipated that the Transition Coordinator role could be easily implemented in various healthcare systems and clinical venues more efficiently than other interventions such as a joint pediatric and adult clinic. This trial will determine whether the support of a Transition Coordinator improves health care and outcomes in young adults with T1D during the transition from pediatric to adult care. Some of the unanswered questions, in part due to the methodological limitations of the existing evidence, have hypothesized that worse outcomes following the transition period are related to patient characteristics rather than a result of the type of care provided. This study is designed to assist in providing a more definitive answer to this question as the randomization should provide comparable groups and remove the allocation bias.

In addition, this study will incorporate the current mandate of the Society for Adolescent Medicine and the recent recommendation of the American Diabetes Association (ADA) for ongoing and expanding research initiatives, emphasizing that “more studies that would examine health outcomes, functional, and long-term outcomes and cost benefit of transition are needed” [1,29,30]. It is anticipated that the uninterrupted, improved quality of diabetes care provided with the support of a Transition Coordinator will result in better glycemic control. Optimizing glycemic control will lead to reduction of diabetes complications, decreased rates of hospitalization, healthcare costs and mortality.

The findings of the current study are expected to support the routine implementation of standardized intervention during the transition period not only in diabetes but also all other areas of care for emerging adults with chronic medical conditions.

## Abbreviations

A1C: Glycosylated hemoglobin; CRO: Contract Research Organization; CDE: Certified diabetes educator; DKA: Diabetic ketoacidosis; ICES: Institute for clinical evaluative studies; JDRF: Juvenile Diabetes Research Foundation; REB: Research Ethics Board; T1D: Type 1 diabetes.

## Competing interests

The authors confirm they have no financial or non-financial competing interests, stocks, shares or patents related to the publication of this manuscript in the past five years.

## Authors’ contributions

TS and CL conceived and designed the study; JLM, JM, IH, ML, EG critically reviewed the design; KE, TR, NB helped develop the role of the Transition Coordinator. All listed authors were involved in the drafting, critical revision and final approval of the version submitted.

## Pre-publication history

The pre-publication history for this paper can be accessed here:

http://www.biomedcentral.com/1471-2431/13/163/prepub
